# Explainable Predictive Model for Suicidal Ideation During COVID-19: Social Media Discourse Study

**DOI:** 10.2196/65434

**Published:** 2025-01-17

**Authors:** Salah Bouktif, Akib Mohi Ud Din Khanday, Ali Ouni

**Affiliations:** 1 Department of Computer Science and Software Engineering College of Information Technology United Arab Emirates University Al Ain, Abu Dhabi United Arab Emirates; 2 Department of Software Engineering and Information Technology École de Technologie Supérieure Montreal, QC Canada

**Keywords:** COVID-19, suicide, social networking sites, deep learning, explainable artificial intelligence, suicidal ideation, artificial intelligence, AI, social media, predictive model, mental health, pandemic, natural language processing, NLP, suicidal thought, deep neural network approach

## Abstract

**Background:**

Studying the impact of COVID-19 on mental health is both compelling and imperative for the health care system’s preparedness development. Discovering how pandemic conditions and governmental strategies and measures have impacted mental health is a challenging task. Mental health issues, such as depression and suicidal tendency, are traditionally explored through psychological battery tests and clinical procedures. To address the stigma associated with mental illness, social media is used to examine language patterns in posts related to suicide. This strategy enhances the comprehension and interpretation of suicidal ideation. Despite easy expression via social media, suicidal thoughts remain sensitive and complex to comprehend and detect. Suicidal ideation captures the new suicidal statements used during the COVID-19 pandemic that represents a different context of expressions.

**Objective:**

In this study, our aim was to detect suicidal ideation by mining textual content extracted from social media by leveraging state-of-the-art natural language processing (NLP) techniques.

**Methods:**

The work was divided into 2 major phases, one to classify suicidal ideation posts and the other to extract factors that cause suicidal ideation. We proposed a hybrid deep learning–based neural network approach (Bidirectional Encoder Representations from Transformers [BERT]+convolutional neural network [CNN]+long short-term memory [LSTM]) to classify suicidal and nonsuicidal posts. Two state-of-the-art deep learning approaches (CNN and LSTM) were combined based on features (terms) selected from term frequency–inverse document frequency (TF-IDF), Word2vec, and BERT. Explainable artificial intelligence (XAI) was used to extract key factors that contribute to suicidal ideation in order to provide a reliable and sustainable solution.

**Results:**

Of 348,110 records, 3154 (0.9%) were selected, resulting in 1338 (42.4%) suicidal and 1816 (57.6%) nonsuicidal instances. The CNN+LSTM+BERT model achieved superior performance, with a precision of 94%, a recall of 95%, an *F_1_*-score of 94%, and an accuracy of 93.65%.

**Conclusions:**

Considering the dynamic nature of suicidal behavior posts, we proposed a fused architecture that captures both localized and generalized contextual information that is important for understanding the language patterns and predict the evolution of suicidal ideation over time. According to Local Interpretable Model-Agnostic Explanations (LIME) and Shapley Additive Explanations (SHAP) XAI algorithms, there was a drift in the features during and before COVID-19. Due to the COVID-19 pandemic, new features have been added, which leads to suicidal tendencies. In the future, strategies need to be developed to combat this deadly disease.

## Introduction

### Overview

Mental health has become a critical area of research as societal challenges, such as pandemics, natural disasters, and economic instability, deeply affect individuals’ well-being. According to comprehensive statistics provided by the World Health Organization (WHO), the occurrence of suicide is documented to be at an average rate of 1 incident every 40 seconds [1]. The report reveals that an estimated 800,000 people die by suicide annually, with suicide attempts occurring at a rate 20 times higher than that of completed suicides [2]. The worldwide approximation for suicide mortality is set at around 1 million deaths per year [3]. These statistics highlight suicide as the primary cause of death among young individuals, particularly among women. Contrary to an “all or nothing” perspective, a well-known book on the subject [4] suggests that suicide follows a distinct process and pattern. The pattern begins with suicidal ideation and progresses toward a suicide attempt and may culminate in a completed suicide. Although not all instances of suicidal ideation lead to complete suicide, they pose a significant threat for individuals to attempt suicide. Recognition of suicidality may occur when individuals frequently discuss it with their caretakers or during interactions with psychiatrists or psychologists who inquire about their thoughts and moods. Caregivers can mitigate the risks associated with suicidality by analyzing various warning signs and implementing necessary preventive measures. However, a significant obstacle in addressing suicidality is the hesitancy of individuals to cooperate with clinicians, often influenced by the social stigma linked to mental illness. The widespread impact of stigma on suicidality affects large-scale clinical interventions for at-risk individuals. Data indicate that 36% of individuals who succumb to suicide leave behind a note [5]. Research suggests that these suicide notes often signal a heightened probability of subsequent attempts that characterize greater precision after an initial failure [6]. The contents of these notes frequently underscore emotions of shame and apology, hinting at a potential willingness to consider alternatives. These notes are typically discovered postcompletion or, at the least, after an attempted suicide [6]. Despite efforts to screen patients, convincing individuals to undergo evaluation remains a formidable challenge in societies marked by stigma [7,8]. Studies highlight that people feel more at ease sharing their day-to-day experiences on online social networks (OSNs), where concerns about social stigma are less prominent [9]. Recent research also suggests that monitoring social media provides an alternative and valuable opportunity to identify warning signs associated with posts from individuals at risk of suicidality [10]. According to the statistics provided by the American Foundation for Suicide Prevention [11], there is a rise in people with mental health conditions. As per the collaborative multiyear project “Suicide Prevention Now” [12], according to statistics, in 2022, there was a significant increase in the number of people reporting mental health conditions. For instance, in 2022, 67% of American believed that they had a mental health condition at some point in their life.

### Related Work and Background Knowledge

Mental health issues, such as depression and suicidal tendency, have traditionally been explored through psychological battery tests and clinical procedures [5,13]. However, to address the stigma associated with mental illness, researchers have turned to less formal platforms, such as social media, to examine language patterns in posts related to suicide. This strategy seeks to enhance the comprehension and interpretation of suicidal ideation. O’Dea et al [14] highlighted the crucial role of questionnaires in assessing a patient’s mental state. However, with the surge in social networking sites, individuals find it more comfortable to openly express their feelings on social media platforms. Studies [13,14] have explored various scales used to predict depression through social media analysis. Additional research [15,16] has examined the topics commonly discussed by potentially suicidal individuals on social media, and the behavior of those contemplating suicide has been scrutinized in these studies. Advances in natural language processing (NLP) and machine learning techniques now allow the extraction of semantic information from social media posts, facilitating the automation of predicting suicidal content [17]. Most research has focused on using binary classification mechanisms with popular algorithms, such as support vector machines (SVMs), decision trees, and ensemble learning algorithms [18-21]. Deep learning methods have also been used to aid in predicting suicidal ideation [22,23]. In Multimedia Appendix 1, we extend the literature review where we explored relevant research works on suicidal ideation prediction (eg, [24-26]).

Feature selection involves identifying and selecting the most relevant attributes or features from the data. We used various feature selection and deep learning techniques, such as term frequency–inverse document frequency (TF-IDF), Word2vec, Bidirectional Encoder Representations from Transformers (BERT), convolutional neural networks (CNNs), and long short-term memory (LSTM). The TF-IDF method involves breaking down text into tokens, calculating the term frequency (TF) to evaluate the significance of each term within a document and determining the inverse document frequency (IDF) to evaluate the uniqueness of each term across the entire data set. Word2vec learns distributed representations (word embeddings) of words based on their context in a given corpus. The resulting word vectors can be used as feature representations for various text-based tasks. Before applying Word2vec, it is essential to preprocess the text data. The Word2vec model is trained on preprocessed text data using either the Skip-gram or the Continuous Bag of Words (CBOW) architecture. We fused deep learning techniques (CNN, LSTM, BERT) in order to effectively identify suicidal ideation as posts with suicidal behavior have a dynamic nature and longer textual segments. BERT is a transformer-based model that uses self-attention mechanisms to capture contextual information. The architecture includes multiple layers of attention and feed-forward networks. BERT provides contextualized embeddings for each token in the input text. In a CNN, input data are a sequence of words or embeddings. The CNN architecture typically involves convolutional layers followed by pooling layers and fully connected layers. A CNN detects important localized features, such as word sequences, n-grams, and syntactic patterns. Each convolutional layer extracts high-level features from the text, which enables the model to capture complex patterns and semantics at different levels of granularity. It also handles input sequences of different post lengths without requiring explicit padding or truncation. LSTM networks are particularly effective for handling sequential data, such as text. LSTM networks are designed to capture long-range dependencies between words and phrases for better understanding of the context and are commonly used in text classification tasks. It propagates information over long distances in the input sequence of data. It processes input texts of different lengths without compromising performance and preserves the sequential structure and context of the posts. The methods are discussed in Multimedia Appendix 2, which provides a theoretical and mathematical background of the deep learning approaches and feature selection methods used in this work.

### Aims of the Study

This work aimed to identify patterns and indicators associated with suicidal ideation by leveraging machine and deep learning algorithms for social media data. The primary focus of this study was to examine the correlation between COVID-19 and the prevalence of suicidal ideation. In this work, various factors were studied related to the pandemic, such as societal, economical, and psychological factors, to detect individuals’ inclination toward suicidal thoughts due to COVID-19. Through comprehensive analysis and empirical investigations, the study sought to find the relationship between COVID-19 and suicidal ideation by detecting potential contributing factors and implications for mental health and well-being. Through rigorous analysis and modeling using deep learning methodologies, this work will contribute by finding the intersection between COVID-19 and suicide, which will be used for developing preventive interventions and support strategies to address future pandemics as well.

The study revolved around the research question of whether COVID-19 has impacted suicidal ideation. The following hypotheses were developed:

Null hypothesis (H0): COVID-19 has not impacted suicidal ideation, and the features (terms) used for suicidal ideation are the same as before.

Hypothesis 1 (H1): COVID-19 has impacted suicidal ideation, and the sequence of terms has changed.

## Methods

### Study Design

**Figure 1:**
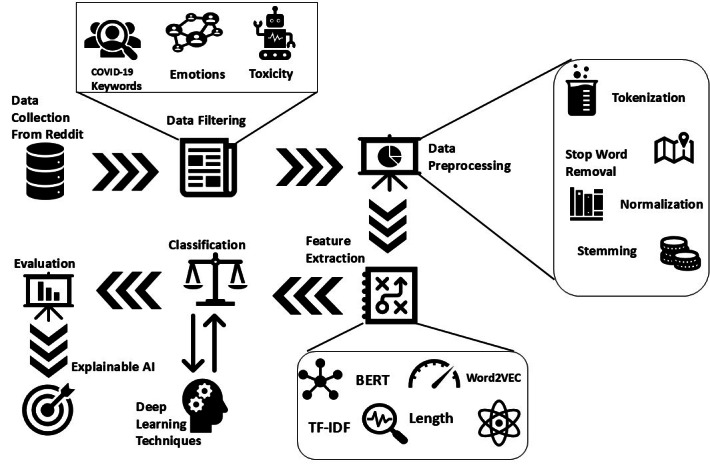
Framework for suicidal ideation detection in the COVID-19 era based on various filtering mechanisms. AI: artificial intelligence; BERT: Bidirectional Encoder Representations from Transformers; TF-IDF: term frequency–inverse document frequency.

This work was divided into 5 phases, starting from data (suicidal posts) collection from Reddit, which is a publicly available data set. In the second phase, the data set was filtered based on various keywords. The emotions of the posts were extracted using the Natural Language Toolkit (NLTK), and the toxicity of the posts was calculated using the perspective application programming interface (API). The final corpus was used for further experimentation, and textual features were extracted using TF-IDF, Word2vec, and BERT techniques. The various classifiers were trained and tested on the filtered data set to classify the posts into 2 classes, suicidal and nonsuicidal. Another aspect of this work was explainable artificial intelligence (XAI); in this phase, 2 techniques, Local Interpretable Model-Agnostic Explanations (LIME) and Shapley Additive Explanations (SHAP), were used to extract the most relevant keywords that trigger suicidal ideation. The study framework is depicted in Figure 1.

### Data Collection and Preparation

Data are the most important entity in all data science research. In this work, there were privacy concerns related to the data. We collected relevant data from the public data repository Kaggle, which contains suicidal posts from the social media platform Reddit. The data were categorized into 2 classes, suicidal and nonsuicidal. The data set consisted of 2 columns, one with the posts (textual) and the other with labels. We leveraged a keyword-based filtering mechanism to classify and analyze user-generated content. We established a comprehensive set of keywords associated with COVID-19 and suicidal ideation, such as “COVID,” “COVID-19,” “lockdown,” “quarantine,” and “coronavirus.” Using these keywords, a dual-layer filtering approach was implemented that categorized the posts into either COVID-19 related or not, followed by an assessment for suicidal content. For further exploration of the data, we performed emotion analysis (EA) to extract the mood of the poster. Eight different emotions were used for EA. After EA, we performed a toxicity analysis (TA) to identify toxic posts, and toxic values were associated with all the posts. We considered only negative, sad, and fearful emotions and posts that had a toxicity value greater than 0.5. In this study, we used the absolute majority (ie, a majority is when more than half of the total weighted words are toxic words). Another alternative was to use two-thirds majority; however, this was not a conservative choice because we risked having a large number of false positives. So, an absolute majority was more protective. By establishing a threshold of 0.5, a clear and straightforward benchmark was created for classifying outputs from our detection models. This threshold simplified both interpretation and implementation for detecting suicidal ideation. It also helped reduce ambiguity in the decision-making process. By doing so, we ensured that texts flagged as indicative of suicidal ideation were those with a probability score of 0.5 or higher. This minimized the risk of misinterpretation as it directed attention to those posts that met a specific criterion for concern. In addition, the empirical accuracies showed that our choice was effective in detecting suicidal ideation. After filtering the posts, we performed data cleaning and preprocessing, in which the textual data was refined using techniques such as tokenization, lemmatization, and normalization. Furthermore, irrelevant data were removed using the stop-word dictionary, and punctuation was also removed.

### Feature Engineering and Selection

The process of selecting the most relevant and informative features from unprocessed data is known as feature engineering, and it increases the machine learning models’ effectiveness [25]. To do this, raw data must be transformed into more significant features that can effectively identify underlying patterns and relationships. This approach makes use of a number of techniques, including feature extraction, feature transformation, and feature selection. Model performance can be greatly influenced by feature engineering through careful feature creation and selection. The data in this study were primarily textual, similar to behavioral analysis of internet users, with the objective of identifying COVID-19–induced suicidal tendencies in online users. To input the data that were collected into the deep learning models, 3 basic word-embedding techniques—TF-IDF [26-28], Word2vec [29], and BERT [30,31]—were used to identify and analyze patterns in the text-based content. Our model leveraged BERT-based architectures. These architectures are designed to capture contextual relationships between words by processing the entire sequence of words simultaneously rather than in isolation. This allows the model to understand the meaning of a word based on its surrounding context, and it significantly improves the accuracy of detecting sarcasm, idiomatic expressions, or shifts in sentiment in languages. The post length is also considered as one of the features. The extracted features were used as input to the CNN and LSTM deep learning models. When used with the CNN and LSTM models, these feature selection techniques improve performance. As mentioned before, CNNs excel at capturing localized patterns in sequential data by making them well suited for text classification, LSTM networks capture long-term dependencies and are effective for modeling sequential data with complex temporal dynamics, and BERT captures contextual relationships between words by processing the entire sequence of words simultaneously.

### Suicide Posts’ Classification

Different classifiers are used for classifying posts into binary classes. In this work, we used multiple combinations of classifiers to predict suicidal posts. The data set we used to train our model consisted of 2 columns, text and label. The problem was formulated the same way as that by Hossain et al [32] and Ji et al [33]. On a corpus consisting of a set of posts 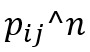 and labels 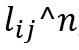, training was provided in such a manner that the model learned from the data consisting of a set of all the engineered features and the corresponding labels that were provided in a supervised approach. The supervisory function is shown in Equation 1:

l_i_ = fun(p_i_) (1)

Here, l_i_ = 1 in the case of p_i_ representing suicide and l_i_ = 0 represents nonsuicide. We focused on the “no free lunch” theorem of machine learning, which suggests that no algorithm can work well for all problems. Three deep learning classification algorithms were implemented: CNN, LSTM, and BERT. These models were fused, as shown in Equation 2:

h_c_ = CNN(X),

h_l_ = LSTM(X),

h_b_ = BERT(X), (2)

h_m_ = concatenate(h_c_, h_l_, h_b_),

y = softmax(Wh_m_ + b),

where X is the input data and is represented as a matrix, where each row corresponds to a data sample and each column corresponds to a feature; h_c_ refers to feature representation by the CNN; h_l_ refers to feature representation by the LSTM; h_b_ refers to feature representation by BERT; h_m_ refers to fused features obtained by concatenating h_c_, h_l_, and h_b_; y is the predicted class probabilities for suicidal posts; and W is the weight of fused features h_m_; and b is the bias of h_m_.

The pseudocode of the entire experiment is shown in the algorithm in Textbox 1. This algorithm outlines the steps for identifying suicidal posts in social media content during the COVID-19 pandemic. The process began with a set of posts and classified them into suicidal and nonsuicidal posts. The algorithm required input posts and ensured the output includes suicidal and nonsuicidal posts. For each post, the algorithm performed EA and TA. Posts were classified based on whether they exhibited sad or negative emotions, alongside a toxicity score above a threshold (0.5). If a post met both criteria, it was added to the final corpus, otherwise it was discarded. The algorithm then processed the remaining posts through tokenization, stop-word removal, and stemming, saving the preprocessed data in a CSV file. Features were extracted using BERT embeddings and TF-IDF, resulting in a document feature matrix. The matrix was trimmed based on minimum document frequencies and TFs to focus on significant terms. Finally, deep learning algorithms and transformers with specific hyperparameters were applied to classify the posts as either suicidal or nonsuicidal. This approach leverages deep learning techniques and NLP to effectively identify suicidal ideation in the context of COVID-19 and ultimately enhances mental health awareness and intervention strategies.

**Pseudocode: deep learning–based suicide behavior identification during COVID-19.**

**REQUIRE:** Posts P

**ENSURE:** Suicidal Post P_s_ and Non-Suicidal Post P_n_

Tokenization → T, StopWordRemoval → SW, Stemming → S, Total Number of Tweets → n

Emotion Analysis → EA , Toxicity Analysis → TA

Term Frequency–Inverse Document Frequency → TF-IDF, Word2vec → w, BERT → B

***START***

1 ***For*** *i from* 1 *to* n

2 Perform EA(P[i])

3 Perform TA(P[i])

4 if (EA(P[i]==Sad/Negative && TA(P[i]>0.5))then

5 C[i]=P[i] // final corpus

6 **Else**

7 Discard

8 ***End For***

9 C[i]=T(C[i])

10 C[i] = SW(C[i])

11 C[i]= S(C[i])

12 Processed.csv= C[i] //Saving Preprocessed file as CSV

13 Features B = B(Processed)

14 Features T=TF/IDF(Processed)

15 Document Feature Matrix=Document Feature Matrix(Features T)

16 Trimmed_dfm=dfm_trim(dfm,min_docfreq,min_termfreq)

17 Final_Features T=Trimmed_dfm.Tfidf //Extracting TFIDF Features

18 Feature w=w(Processed)

19 ***CLASSIFIER*** (Classifier_Name, Hyperparameters, Features)

***END***

### Explainable Artificial Intelligence

XAI holds a central role in mental health by improving the transparency and accountability of artificial intelligence (AI) systems [34,35]. In mental health applications, it is imperative that health care professionals and patients alike grasp and have confidence in the AI’s decision-making procedures [36]. XAI accomplishes this by furnishing explanations for AI-generated diagnoses, forecasts, and treatment recommendations. These explanations provide valuable insights into the rationale behind specific decisions, assisting clinicians and patients in gaining a deeper understanding of the logic behind mental health assessments. This transparency fosters trust and promotes collaboration between AI systems and human experts, which ultimately enhances the quality of mental health care. In addition, XAI aids in detecting and reducing biases within AI models, which ensures that mental health assessments remain impartial and unbiased, a critical aspect of delivering equitable care to diverse patient populations. XAI also contributes to the formulation of individualized treatment plans in the mental health domain. By elucidating factors influencing an individual’s mental health condition, AI systems can customize interventions to cater to each patient’s unique requirements. XAI also facilitates early intervention and prevention by enabling the identification of early warning signs and comprehending the interconnected elements affecting mental well-being. Empowering patients through understandable explanations increases their engagement in the treatment process, potentially leading to better outcomes. XAI serves not only as a tool for enhancing mental health care but also as a means of diminishing stigma by promoting ethical practices and propelling the field of mental health forward through data-driven insights and improved decision-making. XAI techniques are designed to make the decision-making processes of AI systems more understandable and interpretable for humans. These techniques are particularly important when AI systems make complex predictions, classifications, or recommendations in fields such as health care, finance, and autonomous vehicles, where transparency and trust are critical.

LIME generates perturbed instances (z_1_, z_2_,…, z_k_) and performs predictions: f(z_1_), f(z_2_),…, f(z_k_). The local linear model 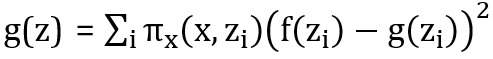. The objective function of LIME is calculated using Equation 3:



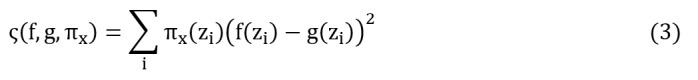



Here, x represents the instance for which we want an explanation, z_i_ are perturbed instances, π_x_(x,z_i_) is a weight, and 
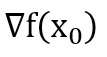
 is the gradient of f at x_0_.

Shapley XAI has the following workings:

The Shapley value for a feature i in the context of a prediction f(x) is shown in Equation 4:



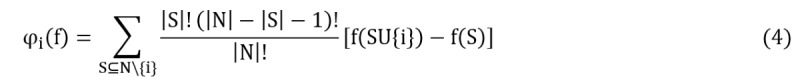



Here, N is the set of all features and S is a subset of features excluding i.

Expected Shapley values are represented in Equation 5:



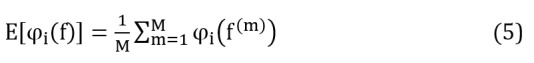



Here, N is the number of samples from the background distribution.

### Ethical Considerations

This research did not include any studies on human participants performed by any of the authors. However, ethical considerations of our study could be related to the use of data collected on posts on the Reddit platform, which is commonly perceived as widely public, where participants are not identifiable as no registration is required but only a pseudo name to post. Moreover, public post analysis, such as Reddit posts, is considered ethical issue free because there is no expectation of privacy as individuals are anonymous, and users understand that their posts can be reused (ie, reposted) and remain archived on the platform until deletion eventually. Hence, ethical issues of research on Reddit are resolved by the public nature of this social media platform, especially when used in academic publications.

## Results

### Study Details

**Figure 2:**
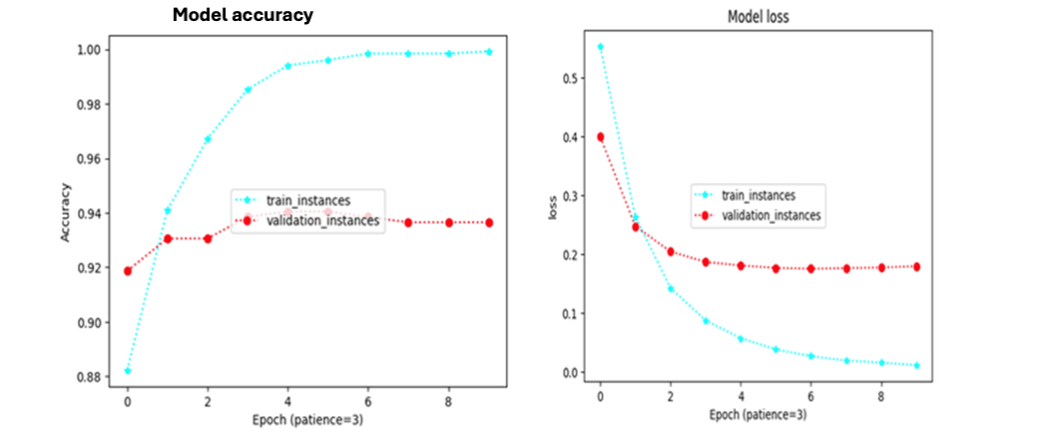
Performance metrics showing the accuracy and loss of the best model, which combined BERT, CNN, and LSTM for enhanced prediction results. BERT: Bidirectional Encoder Representations from Transformers; CNN: convolutional neural network; LSTM: long short-term memory.

Of 348,110 records, 3154 (0.9%) were filtered out, resulting in 1338 (42.4%) suicidal and 1816 (57.6%) nonsuicidal posts. The training data in all experiments were split in an 80:20 ratio for training and validation because empirical findings [37] suggest that the performance of algorithms improves when the data set is divided in this manner. Each algorithm was tested on a distinct subset of the original data set. Our model was evaluated using various metrics to assess their performance. We discovered that the model’s performance improved slightly as the number of training data points was raised. The method of dividing the data set into training and testing subsets has inherent problems with bias and variance. These problems arise because of the nature of the data. In machine learning, it is not necessary that the model that has been fit on training data will also work on real data. This is because real data can be different from training data. To accomplish this, we made use of the technique of k-fold cross-validation. This allowed us to ensure that the model correctly identified the patterns within the data, while minimizing noise exposure. Our model’s validity was established by a process known as 10-fold cross-validation. Python was chosen as the language to implement algorithms for machine learning. Skicit-learn, Pandas, and NLTK are the most significant packages used in the coding process during the training of the suicide prediction model. After evaluating hybrid deep learning models, we found that BERT, when fused with CNN+LSTM, showed the best results among all hybrid deep learning models. With a precision of 94%, a recall of 95%, an *F*_1_-score of 94%, and an overall accuracy of 93.65%. Figure 2 shows the hybrid model’s performance based on the model loss and model accuracy. Table 1 depicts the classification report of all the classifiers that were used during experimentation.

**Table 1:** Classification report of hybrid models based on various evaluation metrics.

Classifier	Precision (%)	Recall (%)	*F*_1_-score (%)	Accuracy (%)
TF-IDF^a^+CNN^b^	76.00	80.00	78.00	78.00
Word2vec+LSTM^c^	88.00	94.00	91.00	88.00
BERT^d^+CNN	69.00	66.00	67.00	64.00
LSTM+BERT	86.00	83.00	85.00	85.00
CNN+LSTM	91.00	90.00	91.00	91.00
BERT+CNN+LSTM	94.00	95.00	94.00	93.65
CNN+LSTM+TFIDF	89.00	89.00	89.00	89.00
CNN+LSTM+Word2vec	90.00	90.00	90.00	90.00
CNN+Word2vec	86.00	86.00	86.00	86.00

^a^TF-IDF: term frequency–inverse document frequency.

^b^CNN: convolutional neural network.

^c^LSTM: long short-term memory.

^d^BERT: Bidirectional Encoder Representations from Transformers.

### Validation

**Figure 3:**
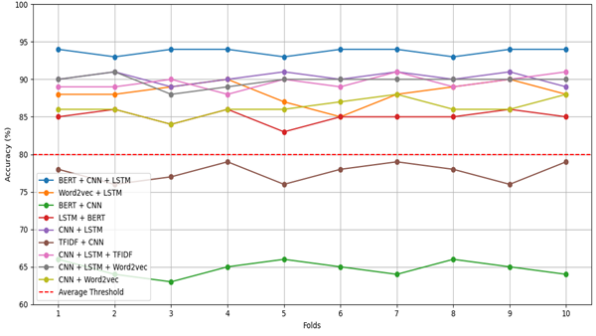
The 10-fold cross-validation process for evaluating model performance through systematic partitioning of the data set. BERT: Bidirectional Encoder Representations from Transformers; CNN: convolutional neural network; LSTM: long short-term memory; TF-IDF: term frequency–inverse document frequency.

In this study, we used the 10-fold cross-validation technique to validate our proposed approach. It is a robust technique frequently used in machine learning and statistical modeling to evaluate the performance and generalization capability of predictive models. In this approach, the data set is divided into 10 equal-size segments, known as folds. Throughout each iteration, 1 fold is designated as the validation set, while the remaining 9 folds serve as the training set. This process is iterated 10 times, with each fold taking on the role of the validation set once. The 10-fold cross-validation technique has the ability to furnish a more dependable assessment of a model’s performance in contrast to a single train-test split. By computing performance metrics across various folds and subsequently averaging them, the ambiguity in outcomes is reduced, which leads to a more consistent evaluation. In addition, it helps in dealing with bias and variance. It guarantees that every data point contributes to both training and validation processes, thereby lessening the risk of overfitting and furnishing a more precise evaluation of the model’s capacity to generalize to new data. In this work, after performing 10-fold cross-validation, the mean accuracy was compared with the actual accuracy of the models, and it was found that there was no such difference. We concluded that the proposed approach does not suffer from over- or underfitting. The results of 10-fold cross-validation are shown in Table 2 and visually represented in Figure 3.

**Table 2:** Results of 10-fold cross-validation, along with the calculated mean accuracy for model evaluation.

Classifier	Accuracy (%)											Accuracy (%), mean (SD)
	Fold 1	Fold 2	Fold 3	Fold 4	Fold 5	Fold 6	Fold 7	Fold 8	Fold 9	Fold 10		
BERT^a^+CNN^b^+LSTM^c^	94.00	93.00	94.00	94.00	93.00	94.00	94.00	93.00	94.00	94.00	93.65 (0.41)	
Word2vec+LSTM	88.00	88.00	89.00	90.00	87.00	85.00	88.00	89.00	90.00	88.00	88.20 (1.58)	
BERT+CNN	66.00	64.00	63.00	65.00	66.00	65.00	64.00	66.00	65.00	64.00	65.00 (1.00)	
LSTM+BERT	85.00	86.00	84.00	86.00	83.00	85.00	85.00	85.00	86.00	85.00	85.00 (0.95)	
CNN+LSTM	90.00	91.00	89.00	90.00	91.00	90.00	91.00	90.00	91.00	89.00	90.20 (0.82)	
TF-IDF^d^+CNN	78.00	76.00	77.00	79.00	76.00	78.00	79.00	78.00	76.00	79.00	77.60 (1.10)	
CNN+LSTM+TF-IDF	89.00	89.00	90.00	88.00	90.00	89.00	91.00	89.00	90.00	91.00	89.60 (0.82)	
CNN+LSTM+Word2vec	90.00	91.00	88.00	89.00	90.00	90.00	90.00	90.00	90.00	90.00	89.80 (0.82)	
CNN+Word2vec	86.00	86.00	84.00	86.00	86.00	87.00	88.00	86.00	86.00	88.00	86.70 (1.10)	

^a^BERT: Bidirectional Encoder Representations from Transformers.

^b^CNN: convolutional neural network.

^c^LSTM: long short-term memory.

^d^TF-IDF: term frequency–inverse document frequency.

### Comparative Analysis of Posts Before and During COVID-19

**Figure 4:**
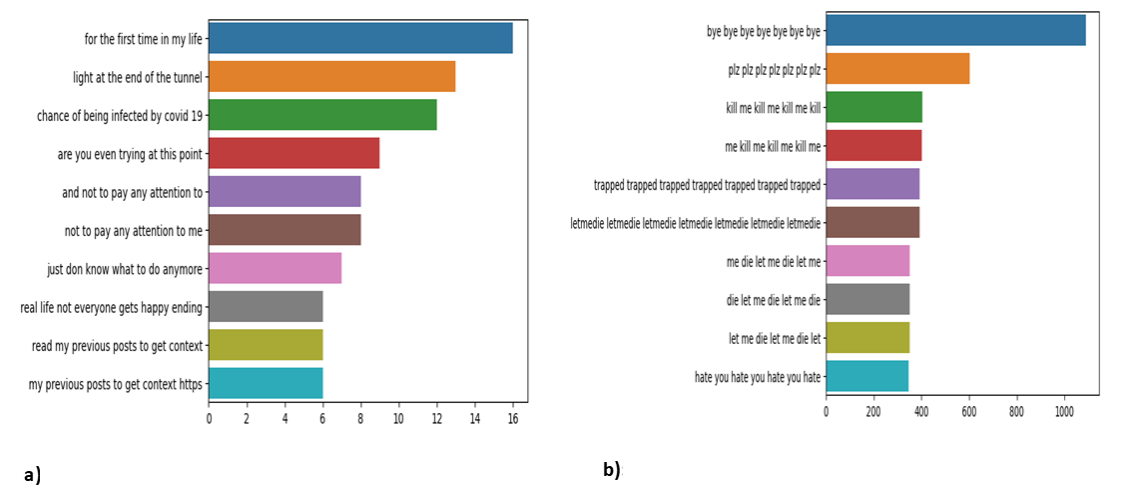
Top n-gram sequences distinguishing COVID-19–related and non–COVID-19–related posts. A) Top n-gram sequence in the COVID-19 suicidal posts; B) Top n-gram sequence in the non COVID-19 suicidal posts.

Exploratory data analysis of both training and validation data sets was performed, and results showed that n-grams used before and during COVID-19 were different. Figure 4 presents a comparative analysis of the n-grams used in suicidal posts during both periods. Figure 4a illustrates the most frequently occurring n-grams in posts related to suicidal ideation during the COVID-19 pandemic, which highlights key phrases and terms that reflect the mental health challenges faced during the crisis, while Figure 4b depicts the n-gram sequences identified in suicidal posts from the non–COVID-19 era.

### Explainable Artificial Intelligence Extraction

**Figure 5:**
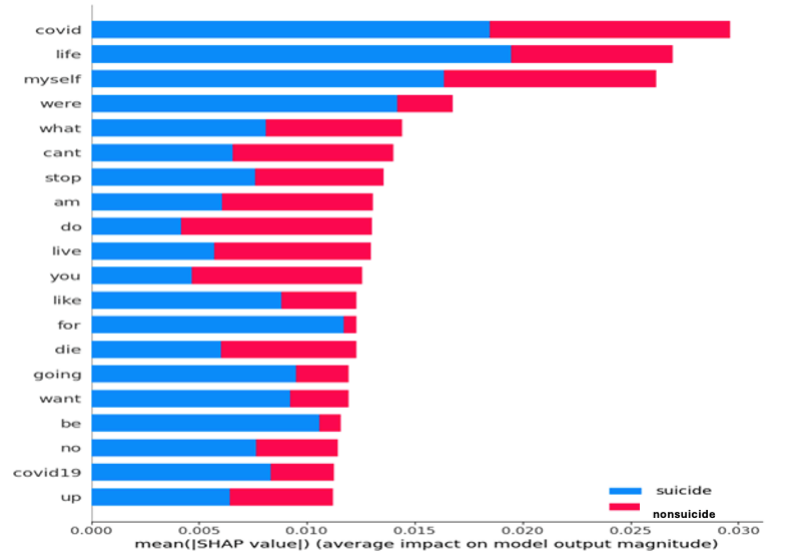
Important features (terms) associated with suicidal posts and their relevance in predictive analysis. SHAP: Shapley Additive Explanations.

XAI showed the most important features (terms) that were used in the suicidal content during COVID-19. Figure 5 shows a graphical representation of the features extracted by XAI:

- “covid,” “covid-19”: These terms indicate a direct association with the COVID-19 pandemic. Individuals may express distress, anxiety, or hopelessness related to the pandemic, which can contribute to suicidal ideation.
- “live, life”: These terms are broad and could encompass various aspects of an individual’s life, such as personal relationships, work, health, or the financial situation. Expressions related to feeling overwhelmed, hopeless, or lacking purpose in life may contribute to suicidal thoughts.
- “die,” “stop,” “myself”: These words directly relate to thoughts of self-harm or suicide. They indicate a strong presence of negative thoughts or intentions toward ending one’s life.

XAI was used to check the impact of COVID-19 on suicidal ideation, and it can be clearly seen from the results that COVID-19–related words were used frequently in suicidal thoughts.

## Discussion

### Principal Findings

The results of this study indicate significant changes in language patterns before and during the COVID-19 pandemic. Individuals used different sets of words prior to the pandemic, and the sequence of words shifted during the pandemic. In this work, we investigated the impact of the COVID-19 pandemic on suicidal ideation by proposing a null hypothesis (H_0_) that COVID-19 has not influenced levels of suicidal thoughts among individuals. By using the proposed methodology, our findings revealed a significant increase in suicidal ideation associated with the pandemic. The analysis showed a *P* value of <.01, which leads us to reject H_0_ in favor of the alternative hypothesis (H_1_), which posited that COVID-19 has had a measurable impact on suicidal ideation. These results underscore the urgent need for mental health interventions during and after public health crises, highlighting the critical link between worldwide events and individual psychological well-being.

### Comparative Analysis

For validating the approach, we performed comparative analysis in which we compared existing methods with our approach. The methodology described by Jung et al [38] showed that Bow-Char had a maximum recall score of 0.75, closely trailed TF-IDF (SVM) with a recall of 0.70. In our approach, the recall scores ranged from 0.83 to 0.95, which are higher than existing ones. The results demonstrated that the BERT+CNN+LSTM model had the highest recall, proving its efficacy in identifying positive instances. In addition, ADA achieved the higher precision of 0.92 compared to other approaches that were proposed by Jung et al [38], while our approach achieved precision values from 0.86 to 0.95. The BERT+CNN+LSTM model showed that it can minimize false positives by attaining the maximum level of accuracy. The range of accuracy attained by Jung et al [38] was 65%-77%. Our proposed models, in contrast, outperformed them with accuracy scores between 0.85 and 0.9365. The best accuracy was shown by the BERT+CNN+LSTM model, demonstrating its capacity to classify most cases properly. According to Saba et al [39], a recurrent neural network (RNN) performs better due to its high precision value (1), while CNN models achieve good recall values of 0.8 and CNN+SVM models achieve 91.6% accuracy. Our approach outperformed previous methods by achieving greater recall, precision, and accuracy values; the BERT+CNN+LSTM model achieved 93.65% accuracy. These findings show that the proposed method produces more accurate and reliable results.

When *F*_1_-scores were examined, ADA had the highest score of 0.73 among the techniques examined by Jung et al [38], while the RNN had an *F*_1_-score of 0.76 and the CNN and CNN+SVM models had the highest *F*_1_-scores of 0.85 and 0.90 according to Saba et al [39]. Our approach outperformed in *F*_1_-scores as well by achieving values ranging from 0.85 to 0.94. The best *F*_1_-score was attained by the BERT+CNN+LSTM model, demonstrating its balanced precision and recall performance. The TF-IDF+CNN model outperformed Jung et al’s [38] best method in terms of recall by about 20%. Comparably, the CNN+LSTM and CNN+LSTM+Word2vec models outperformed Jung et al’s [38] optimal approach by about 15% and 14%, respectively, in terms of recall values. These notable advancements suggest that the proposed techniques are better at identifying positive samples. The accuracy of the TF-IDF+CNN model was around 21.65% greater than that of Jung et al’s [38] optimal method (TF-IDF+SVM). Similarly, accuracy gains of roughly 19% and 18% were attained by our CNN+LSTM and CNN+LSTM+Word2vec models, respectively. These improvements show that the proposed approaches have stronger categorization skills and produce results that are more accurate. Hence, we can conclude that the recall, precision, accuracy, and *F*_1_-score of the proposed methods—TF-IDF+CNN, BERT+LSTM, CNN+LSTM, and Word2vec+CNN+LSTM—are better than existing techniques. The enhancements in performance range from 14% to 21%, highlighting the efficacy of using sophisticated methods, such as TF-IDF, CNNs, LSTM, and BERT, when examining content.

### Limitations

We analyzed the strengths and weaknesses of each model, and it was found that the CNN effectively captured localized patterns in social media posts, such as emotional language and contextual cues indicative of suicidal ideation. Phrases such as “I feel hopeless” and “No one cares” were accurately classified as indicating suicidal ideation. However, the model struggled with understanding context, particularly when the meaning of a post depended heavily on the sequence of words rather than their individual presence. For example, the phrase “I just need a break” was incorrectly classified as nonsuicidal. In contrast, the LSTM processed sequences that made it suitable for understanding the context and flow of language over time. For example, posts beginning with “Things have been tough lately” and continued with “I can’t see a way out” were correctly identified by the LSTM as having an evolving sentiment of despair and resulted in the correct classification of suicidal ideation. However, there is a risk of overfitting to the training data, especially if the training set is small or not diverse enough. Although the LSTM captured the emotional tone, it may have overfit to similar phrases in the training set that did not indicate suicidal ideation and resulted in false negatives. For example, “I can't handle this anymore, I'm just tired of fighting” was misclassified as nonsuicidal. BERT processes text in both directions simultaneously. This allows it to capture the full context of a word based on its surroundings, leading to better understanding and representation of meaning, but it benefits from being trained on large data sets. Since we had less data, we integrated the three algorithms (BERT, CNN, and LSTM) in our hybrid model, which helped us mitigate the individual weaknesses of each model. The CNN effectively extracted features from social media posts, the LSTM provided a contextual understanding of the text, and BERT considered the entire context of each word, which effectively disambiguated words with multiple meanings. Combining these architectures allowed us to capture both localized and generalized contextual information that is crucial for understanding the language patterns associated with suicidal behavior. By fusing these 3, our model achieved an accuracy of 93.65%.

### Implications of the Study

The findings of this study reveal that the COVID-19 pandemic has influenced mental health. Due to the shift in language patterns, there is an increase in terms related to distress, hopelessness, and self-harm, which underscores the profound psychological impact of the pandemic. A clearer understanding of linguistic markers that may indicate heightened risk can be identified using XAI. These linguistic patterns have broader implications for early detection and intervention strategies for suicidal ideation. We suggest that language-based monitoring could serve as a tool for identifying individuals at risk. In addition, the study emphasizes the need for targeted mental health support during worldwide crises because the psychological effects of such events can lead to a lasting transformation in how individuals express their struggles. The findings suggest a critical shift toward more personalized language-based approaches to mental health care that can adapt to the unique challenges posed by worldwide events, such as the COVID-19 pandemic.

### Conclusion

The worldwide impact of COVID-19 has had long-lasting effects on people’s mental well-being. In this study, we collected data from Reddit posts related to COVID-19. Data were filtered using specific keywords associated with the pandemic. Through EA and TA, we understood the emotional tone and potential harm in these posts. To classify the data, hybrid deep learning classifiers were used, which fused BERT, CNN, and LSTM models. The results reveal that our BERT+CNN+LSTM model achieved the highest accuracy of 93.65%, with a precision of 94% and a recall of 95%. In the final stage, XAI techniques, such as LIME and SHAP, extracted important factors for suicidal and nonsuicidal sentiments. After integration, the fused model can effectively grasp both localized and generalized contextual characteristics that are essential for comprehending linguistic patterns linked with suicidal ideation. It manages diverse formats commonly encountered in real-world text data by accommodating varying sentence lengths and syntactic complexities. This model will equip decision makers with a deeper understanding of factors contributing to suicide risk, facilitating the development of more impactful intervention strategies. Our analysis showed that terms related to COVID-19 are often present in posts with suicidal tendencies. We plan to incorporate audio data alongside these features to enhance the prediction of suicidal ideation.

## Appendix

Multimedia Appendix 1 Detailed literature.

Multimedia Appendix 2 Background knowledge of various methods used.

## Abbreviations

AI: artificial intelligence
BERT: Bidirectional Encoder Representations from Transformers
CNN: convolutional neural network
EA: emotion analysis
LIME: Local Interpretable Model-Agnostic Explanations
LSTM: long short-term memory
NLP: natural language processing
NLTK: Natural Language Toolkit
SHAP: Shapley Additive Explanations
SVM: support vector machine
TA: toxicity analysis
TF-IDF: term frequency–inverse document frequency
XAI: explainable artificial intelligence

## Data Availability

The data used in this study are available upon request from the corresponding author.
