# Supplementary material for: Explainable Predictive Model for Suicidal Ideation During COVID-19: Social Media Discourse Study
Source: J Med Internet Res. 2025 Jan 17;27:e65434. doi: 10.2196/65434 (PMC11786132; doi:10.2196/65434)
Supplement: Multimedia Appendix 1 [file jmir_v27i1e65434_app1.docx]

The review of existing literature revealed a significant gap in the field of data mining concerning the prediction of suicidal ideation on social media and its prevention. This gap suggests a need for substantial efforts in research. Also, the scarcity of data poses a considerable challenge, primarily due to privacy and ethical concerns related to this area of study. Table S1 summarizes the recent work in suicidal ideation detection. The available literature primarily concentrates on binary classification and employs conventional feature extraction techniques. In contrast, our work represents a novel approach by gathering extensive data on suicidal context on Reddit. Moreover, our focus extends to developing a feature extraction mechanism to capture rich features from the data.

**Table S1.** Most relevant and recent work related to the detection of suicidal ideation on social media.

| **Title of the Paper** | **Contributions** | **Methods/Techniques** | **Limitations** |
| --- | --- | --- | --- |
| “Feeling bad on facebook: Depression disclosures by College Students on a Social Networking Site [1]” | Evaluating Facebook Status of College Students for Depression and Major Depressive Episode | Negative Binomial Regression model and SATA is used. | Limited dataset. |
| “Detecting suicidality on Twitter [2]” | Developed a Machine Learning Classifier for suicide detection. | SVM and LR. | Less suicide-related keywords were used for extraction and Traditional feature mechanism was used. |
| “Analysing the connectivity and communication of suicidal users on Twitter [3]” | To find the common links between suicide ideation users. | Social graphs. | More than one-hop away neighbours and demographic characteristics can be used. |
| “Multi-class Machine classification of suicide-related communication on Twitter [4]” | Machine Learning Classifier. | DT, SVM and NB. | Only Twitter data used and features are not explored. |
| “A Survey on Prediction of Suicidal Ideation Using Machine and Ensemble Learning [5]” | Machine Learning and Ensemble Learning Models proposed for Suicidal Ideation. | Logistic Regression, Bernoulli Naïve Bayes, Multinomial Naïve Bayes, Decision Tree, Random Forest, Support Vector Machine, and AdaBoost. | Precision value of less than 50% and a recall value of less than 50%, indicating false positive and false negative alarms. |
| “A Computational Approach to Feature Extraction for Identification of Suicidal Ideation in Tweets [6].” | The study focused to develop and design features that can help in distinguishing suicidal and non-suicidal tweets. | Random forest and Hybrid features. | Only Twitter data used and exploration of data is missing. |

The following conclusions can be drawn from the literature survey.

- The majority of study in this field has been focused on machine learning and its optimization, with comparatively less attention paid to deep learning studies and their implementation.
- Most of the research work has gone into improving and fine-tuning classifiers, with little focus on the important steps of preprocessing and data preparation, which are essential to ensuring the accuracy and dependability of the outcomes.
- From the literature survey it is evident that there hasn't been much thorough investigation or application of feature engineering methods to improve the system's overall efficacy and performance.

## References

1. Moreno MA, Jelenchick LA, Egan KG, Cox E, Young H, Gannon KE, Becker T. Feeling bad on Facebook: depression disclosures by college students on a social networking site. *Depress Anxiety* 2011 Jun 11; 28(6):447-455.
2. O'Dea B, Wan S, Batterham PJ, Calear AL, Paris C, Christensen H. Detecting suicidality on Twitter. *Internet Interven* 2015 May; 2(2):183-188.
3. Colombo GB, Burnap P, Hodorog A, Scourfield J. Analysing the connectivity and communication of suicidal users on Twitter. *Comput Commun* 2016 Jan 01; 73(Pt B):291-300.
4. Burnap P, Colombo G, Amery R, Hodorog A, Scourfield J. Multi-class machine classification of suicide-related communication on Twitter. *Online Soc Netw Media* 2017 Aug; 2:32-44.
5. Chadha A, Kaushik B. A survey on prediction of suicidal ideation using machine and ensemble learning. *Comput J* 2021; 64(11):1617-1632.
6. Sawhney R, Manchanda P, Singh R, Aggarwal S. A computational approach to feature extraction for identification of suicidal ideation in tweets. 2018. Presented at: ACL 2018, Student Research Workshop; July 15-20, 2018; Melbourne, Australia. p. 91-98.
