# Supplementary material for: Explainable Predictive Model for Suicidal Ideation During COVID-19: Social Media Discourse Study
Source: J Med Internet Res. 2025 Jan 17;27:e65434. doi: 10.2196/65434 (PMC11786132; doi:10.2196/65434)
Supplement: Multimedia Appendix 2 [file jmir_v27i1e65434_app2.docx]

### TF/IDF

The TF/IDF is utilized to analyze textual data. The TF/IDF method involves breaking down the text into tokens, calculating the term frequency (TF) to evaluate the significance of each term within a document, and determining the inverse document frequency (IDF) to evaluate the uniqueness of each term across the entire dataset. The term frequency of a term $t$in a document $d$is calculated as:

$$\mathrm{TF}\left( t,d \right)=\frac{Number of times term t appears in document d}{Total number of terms in document d}$$

The inverse document frequency of a term $t$ in a collection of documents $D$is calculated as:

$$\mathrm{IDF}\left( t,D \right)=\log\left( \frac{Total Number of Documents in D}{Documents containing term t} \right)+1$$

The TF-IDF score for a term $t$ in a document $d$ within a collection $D$is calculated as the product of TF and IDF:

$$TF-IDF\left( t,d,D \right)=TF\left( t,d \right)*IDF\left( t,D \right)$$

This method is used when looking at text that is related to suicide. Combining these measurements determines TF/IDF weight for each phrase that appears in every document. The TF/IDF matrix that was created as a result acts as a set of features, depicting each document as a vector of terms with varying degrees of importance. This tactic assists in the identification of phrases that are both prevalent inside a document and distinctive across the entirety of the body of text, providing insights into important themes related to suicide

### Word2vec

Word2Vec is a popular technique for feature engineering in natural language processing. It learns distributed representations (word embeddings) of words based on their context in a given corpus. The resulting word vectors can be used as feature representations for various text-based tasks. Before applying Word2Vec, it's essential to preprocess the text data. The Word2Vec model is trained on the preprocessed text data using either the Skip-gram or Continuous Bag of Words(CBOW) architecture. The model parameters include:

- **Embedding Size:** The dimensionality of the word vectors (e.g., 100, 200).
- **Context Window:** The number of surrounding words considered during training.

The training objective is to maximize the likelihood of predicting context words given a target word (Skip-gram) or predicting a target word given its context.

In the Skip-gram model the objective is to maximize the probability and is calculated by Equation 1.

$$\prod_{t=1}^{T} \prod_{j=-c}^{c} P\left( w_{t+j} | w_{t} \right) 1$$

where:

- T is the total number of words in the corpus.
- c is the context window size.
- P(w_t+j_ | w_t_) is the conditional probability of the context word given the target word.

In CBOW model the objective is to maximize the probability, which is calculated by Equation 2.

$$\prod_{t=1}^{T} P(w_{t}|\mathrm{context}\left( w_{t} \right)) 2$$

where *context(w_t_)* represents the context words around *w_t_.*

Once the Word2Vec model is trained, each word in the vocabulary is represented as a dense vector in the embedding space. These vectors capture semantic relationships between words. For example:

Word vector for *"king" = [0.2, -0.4, 0.7, …]* (100-dimensional vector)

Word vectors obtained from Word2Vec serve as feature representations for text. In a given document, the feature vector can be generated by aggregating the word vectors of constituent words. The resulting feature vectors are used as input representations for suicidal ideation text classification.

### BERT

BERT (Bidirectional Encoder Representations from Transformers) is a pre-trained transformer model designed for natural language understanding tasks. It can be used for feature extraction and classification also. Before applying BERT for feature extraction, the text data needs to be preprocessed. BERT is a transformer-based model that utilizes self-attention mechanisms to capture contextual information. The architecture includes multiple layers of attention and feed-forward networks. BERT provides contextualized embeddings for each token in the input text. The final hidden states from the model can be used as features. For a given input sequence *X*, BERT produces a sequence of hidden states *H = {h_1, h_2, ..., h_n_},* where *n* is the number of tokens.The features for each token can be extracted by Equation 3.

$$\mathrm{Features}\left( X \right)=\left\{ h_{1},h_{2},\ldots, h_{n} \right\} 3$$

For classification tasks, often the hidden state corresponding to the [CLS] token is used as the aggregated representation of the entire sequence.

*Aggregated Features(X) = h_[CLS]_*

**Example Sentence**: "Akib is a dedicated and hardworking employee, but his communication skills need improvement."

- Tokenize the sentence into individual words:

*"Akib", "is", "a", "dedicated", "and", "hardworking", "employee", ",", "but", "his", "communication", "skills", "need", "improvement", "."*

- Convert the tokens into BERT-compatible input format:

*"[CLS]", "Akib", "is", "a", "dedicated", "and", "hardworking", "employee", ",", "but", "his", "communication", "skills", "need", "improvement", ".", "[SEP]"*

- Adding special tokens ([CLS] and [SEP]) and padding the sequence to a fixed length.
- **BERT embeddings :** "Akib" 🡪[0.56, 0.72, -0.39, ...]
- **Dimensional Reduction of BERT embeddings:** [0.32, -0.18, 0.67, ..., 0.45, -0.27, 0.13]
- **Feature Importance Scores.** "Akib" 🡪0.58}
- **Selecting the most important embeddings as inputs.** "Akib" 🡪0.58, "communication" 🡪-0.42}

### CNN

A Convolutional Neural Network (CNN) can be utilized for text classification tasks, where the input data is a sequence of words or embeddings. The architecture typically involves convolutional layers followed by pooling layers and fully connected layers.

Let *X* be the input text data represented as a sequence of word embeddings or one-hot encoded vectors. The input can be denoted as Equation 4:

$$X=\left\{ x_{1},x_{2},\ldots, x_{n} \right\} 4$$

where *n* is the length of the sequence. Applying convolutional operations to capture local patterns and features in the input sequence. The convolution operation can be represented as Equation 5:

$$c_{i}=\sigma\left( W_{i}*x+b_{i} \right) 5$$

where *W_i_* is the filter, *x* is the input, bi is the bias term, and $\sigma$ is the activation function.

Applying pooling layers (e.g., max pooling) to reduce the spatial dimensions and retain the most important features. For a given feature map $c_{i}$, max pooling can be denoted as Equation 6:

$$p_{i}=\max\left( c_{i} \right) 6$$

Flatten the pooled features and pass them through fully connected layers to make predictions. The fully connected operation can be expressed as Equation 7.

$$y=soft\max\left( W_{fc}*p+b_{fc} \right) 7$$

where $W_{fc}$is the weight matrix, *p* is the flattened pooled feature, $b_{fc}$ is the bias term, and *softmax* is the activation function.

The training objective involves minimizing a suitable loss function, such as cross-entropy, which can be represented as Equation 8.

$$\mathrm{Loss}=-\sum_{i} y_{i}\log\left( {y`}_{i} \right) 8$$

where $y_{i}$is the true class probability and ${y`}_{i}$ is the predicted class probability.

### LSTM

Long Short-Term Memory (LSTM) networks are a type of Recurrent Neural Network (RNN) that are particularly effective for handling sequential data, such as text. LSTMs are designed to capture long-range dependencies and are commonly used in text classification tasks. Let X be the input text data represented as a sequence of word embeddings or one-hot encoded vectors. The input sequence can be denoted as Equation 9:

$$X=\left\{ x_{1},x_{2},\ldots, x_{n} \right\} 9$$

where *n* is the length of the sequence.

The LSTM cell consists of three gates: an input gate *i_t_,* a forget gate *f_t_,* and an output gate *o_t_.* The computation within an LSTM cell can be expressed as:

$$i_{t}=\sigma(W_{ii} . x_{t}+b_{ii}+W_{hi} . h_{t-1}+b_{hi})$$

$$f_{t}=\sigma(W_{if} . x_{t}+b_{if}+W_{hf} . h_{t-1}+b_{hf})$$

$$o_{t}=\sigma(W_{io} . x_{t}+b_{io}+W_{ho} . h_{t-1}+b_{ho})$$

$$c_{t}=\tanh(W_{ig} . x_{t}+b_{ig}+W_{hg} . h_{t-1}+b_{hg})$$

$$c_{1}=f_{t} . c_{t-1}+i_{t}. c_{t}$$

$$h_{t}=o_{t}.tanh(c_{t})$$

Here, $x_{t}$ is the input at time step *t,* $h_{t-1}$ is the hidden state from the previous time step, $c_{t-1}$ is the cell state from the previous time step, and $W$ and $b$ are weight matrices and bias vectors, respectively.

The output of the LSTM sequence is typically passed through a fully connected layer to obtain the final classification result as shown in Equation 10.

$$y=\mathrm{softmax}\left( W_{fc} . h_{n}+b_{fc} \right) 10$$

where $W_{fc}$ is the weight matrix, $h_{n}$is the final hidden state, $b_{fc}$ is the bias term, and $\mathrm{softmax}$ is the activation function.

The training objective involves minimizing a suitable loss function, such as cross-entropy, which can be calculated as Equation 11.

$$\mathrm{Loss}=-\sum_{i} y_{i}\log\left( {y`}_{i} \right) 11$$

where $y_{i}$ is the true class probability and ${y`}_{i}$ is the predicted class probability.
